# The prevalence of increased serum IgE and *Aspergillus* sensitization in patients with COPD and their association with symptoms and lung function

**DOI:** 10.1186/s12931-014-0130-1

**Published:** 2014-10-29

**Authors:** Jianmin Jin, Xiaofang Liu, Yongchang Sun

**Affiliations:** Department of Respiratory Medicine, Beijing Tongren Hospital, Capital Medical University, Beijing, China

**Keywords:** Immunoglobulin E, Allergy, Chronic obstructive pulmonary disease, *Aspergillus*

## Abstract

**Background:**

Allergy and *Aspergillus* hypersensitivity (AH) were shown to be associated with severe symptoms or worse lung function in COPD patients. The prevalence of elevated total IgE (T-IgE) and its association with clinical symptoms and lung function in COPD have not been studied. The prevalence of AH and its correlation with clinical characteristics in a COPD cohort of larger sample size is also lacking.

**Methods:**

273 patients with COPD were evaluated by respiratory symptoms, blood test, chest HRCT, lung function, serum detection of T-IgE and *Aspergillus* specific IgE. Patients with T-IgE ≥ 1000 KU/L were further investigated for allergic bronchopulmonary aspergillosis (ABPA).

**Results:**

The prevalence of elevated T-IgE and AH in patients with COPD was 47.3% and 15.0%, respectively. Eight patients (2.9%) met the diagnostic criteria for ABPA. Compared with the normal T-IgE group, patients with elevated T-IgE had a longer history of dyspnea (p < 0.01), an earlier onset of dyspnea after chronic cough/expectoration (p < 0.01), and were more likely to wheeze (p < 0.01). They also showed worse lung functions and more severe GOLD staging (p < 0.01). Analysis of the clinical data in male patients with smoking as the risk factor showed the same results. To evaluate the clinical characteristics of COPD with AH, patients with elevated T-IgE were further divided into subgroups with and without AH. When compared with the normal T-IgE group, both the two subgroups showed longer history of dyspnea (p < 0.01), an earlier onset of dyspnea (p < 0.01) and a worse status of lung function (p < 0.05). Correlation analysis demonstrated that T-IgE was correlated positively with the time length of dyspnea (r = 0.401, p < 0.001), and the ratio of duration of dyspnea to that of chronic cough/expectoration (r = 0.59, p < 0.001), but negatively with FEV1/FVC% (r = −0.194, p = 0.001), and FEV1%predicted (r = −0.219, p < 0.001).

**Conclusions:**

There was a high prevalence of elevated serum T-IgE and AH in patients with COPD. Serum T-IgE level was correlated with symptoms such as dyspnea and impairment of lung function. Allergens other than *Aspergillus* may have similar effects on disease expression or progression of COPD.

## Background

Chronic obstructive pulmonary disease (COPD) is characterized by persistent airflow limitation, and is a major cause of morbidity and mortality worldwide. COPD is a heterogeneous disease and can be classified into different “phenotypes” [1]. A recent study by Jamieson et al. [2] showed that there was an “allergic phenotype” of COPD, which accounted for 21% or 30% by allergy history (doctor-diagnosed hay fever or allergic symptoms) or allergy testing (increased allergen-specific IgE) in two cohorts. Patients with the allergic phenotype of COPD had more and severe respiratory symptoms and frequent exacerbations, although no significant difference in lung function compared to the non-allergic phenotype [2]. Of the common specific allergens, *Aspergillus fumigatus (A. fumigatus)* sensitization is of clinical importance in both asthma and COPD. The prevalence of *Aspergillus* hypersensitivity (AH) and allergic bronchopulmonary aspergillosis (ABPA) in asthma was 28% and 12.9% respectively in one study [3]. An investigation from Agarwal et al. [4] found that the prevalence of AH, defined by the presence of immediate cutaneous hyperreactivity to the aspergillus antigen, and ABPA in COPD without obvious atopy was 8.5% and 1% respectively. More recently Bafadhel et al. [5] demonstrated that AH was present in 13% of COPD subjects and was associated with worse lung function.

In the study by Jamieson et al. [2], the “allergic phenotype” of COPD was determined in two different cohorts by a history of mucosal allergy or positive serum specific IgE. However, some patients with asthma or coexisting COPD and asthma could not be excluded from the study [2]. In addition, it is highly probable that detecting only some of the common allergens cannot fully reflect the allergic condition of a patient, since there are so many kinds of potential allergens in the environments and some are unknown to humans. In this case, the serum total IgE (T-IgE) may be a more sensitive marker for hypersensitive condition of the host. Meanwhile, it is possible that serum IgE, which was shown to be related to airway inflammation and remodeling in asthma [6-9], may have effects on symptoms and lung function of COPD. Until now, data on the allergic status of COPD patients and its association with clinical symptoms and lung function are scarce. Therefore in a single center and cross-sectional study, we examined the prevalence of increased serum T-IgE, *A. fumigatus* sensitization and ABPA in patients with COPD. We hypothesized that compared with the nonallergic patients, patients with increased serum T-IgE and/or *A. fumigatus* sensitization may have more severe or longer history of respiratory symptoms, and worse lung functions.

## Subjects and methods

### Subjects and diagnostic process

**Figure 1:**
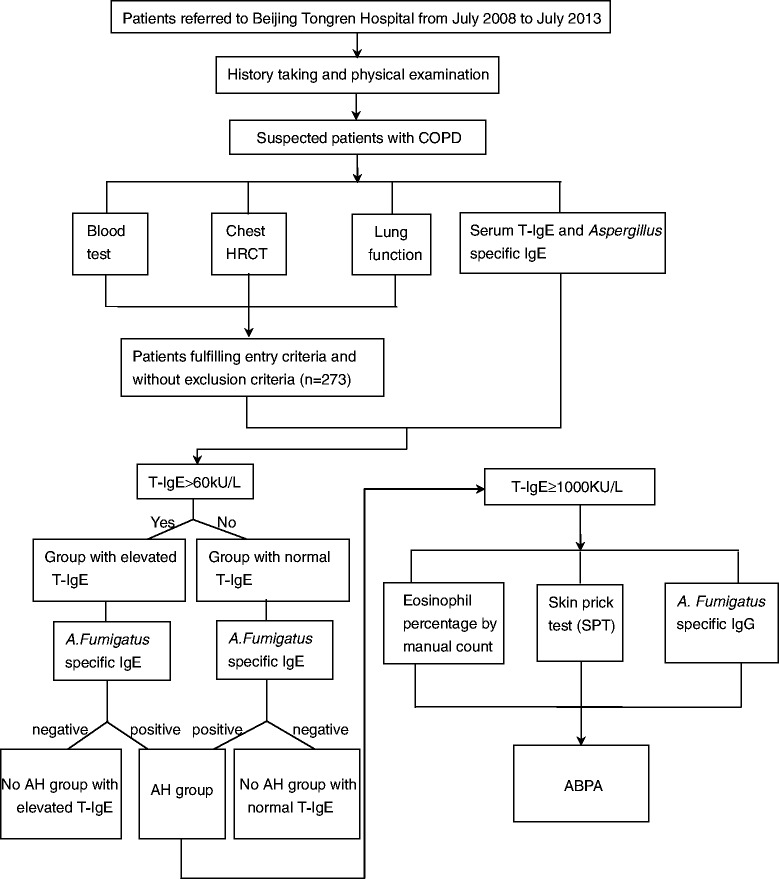
**Selection and diagnostic procedure of study subjects.** *Definition of abbreviations*: HRCT: high resolution computed tomography, T-IgE: serum total IgE, *A. Fumigatus*: *Aspergillus Fumigatus*, AH: *Aspergillus* hypersensitivity, ABPA: Allergic bronchopulmonary aspergillosis.

Patients with COPD visiting Beijing Tongren Hospital from July 2008 to July 2013 were enrolled. The subject selection and diagnostic process were shown in Figure 1. The study was approved by the local ethics committee of Beijing Tongren Hospital, Capital Medical University, and written informed consent was obtained from all patients.

To exclude the possible effects of gender and risk factors on the symptoms and lung function of COPD patients, male patients with smoking history but no exposure to biomass fuel and occupational dusts were divided into two groups according to the serum level of T-IgE, and patients with elevated T-IgE were further divided into two subgroups according to whether hypersensitive to *Aspergillus*.

Serum samples from 150 age- and gender-matched volunteers (as the control population) without history of doctor-diagnosed allergy (such as allergic rhinitis and eczema) or chronic airway diseases (such as COPD, asthma and chronic bronchitis) were collected for detection of T-IgE.

The patients were diagnosed to have COPD by the following criteria: (i) age more than 40 years, (ii) chronic cough, expectoration and/or wheeze for at least three months in a year for two successive years, (iii) current or history of smoking (smoking index ≥10 pack-year), and/or history of exposure to biomass fuel for at least 10 years, and/or history of occupational exposure to noxious particles and fumes for at least 10 years, and (iv) evidence of irreversible obstructive impairment on spirometry defined by postbronchodilator FEV1/FVC <70%. The enrolled patients were required to have no history of doctor-diagnosed allergy such as allergic rhinitis and eczema, no obvious food allergy, no family history of asthma, and no evidence of parasite infection. The enrolled patients with COPD should have no acute exacerbation of COPD [10] within at least 2 weeks. Patients were excluded from the study if they met any of the following criteria: (i) receiving systemic steroid therapy in the preceding 4 weeks, (ii) receiving any other immunosuppressive therapy, (iii) with active pulmonary tuberculosis, interstitial lung disease, and severe heart failure.

Definition of respiratory symptoms: chronic cough and expectoration were defined as present if reported for three or more consecutive months. Wheeze was considered present if the patient experienced spasmodic dyspnea from wheezing or “whistling” in the chest. Exertional dyspnea was identified as present if the mMRC (Modified Medical Research Council Questionnaire) score was equal to or more than 2 [11]. The duration of history of a symptom, for example, the duration of dyspnea history, was defined as the time length from the onset of the symptom to enrollment in the study. As in patients with COPD, chronic cough and expectoration generally precedes exertional dyspnea for a different period of time, and dyspnea is a major cause of disability and anxiety associated with the disease, we used the ratio of duration of dyspnea history to that of chronic cough/expectoration history to indicate the relative onset of dyspnea during the chronic course of the disease.

*A. fumigatus* sensitization was defined if an elevated serum level of specific IgE (>0.35 kUA/L) was detected. Diagnosis of ABPA was made according to the Patterson criteria [12], fulfilling at least six of the following eight criteria: (i) asthma (episode of bronchial obstruction), (ii) positive result of skin prick test (SPT) for *A. fumigatus*, (iii) elevated serum total IgE (≥1000 kU/L), (iv) elevated serum *A. fumigatus* specific IgE (>0.35 kUA/L), (v) elevated IgG antibodies in serum against *A. fumigatus*, (vi) eosinophilia (the eosinophil percentage of peripheral blood ≥5%), (vii) central bronchiectasis, (viii) transient or fixed pulmonary opacities. The diagnosis of ABPA with central bronchiectasis (ABPA-CB) and that without (seropositive form, ABPA-S) were made based on lung HRCT findings as previously described [13].

### Detection of serum T-IgE, *A. fumigatus* specific IgE and IgG

An automatic immunoassay system (ImmunoCap TM 100, Pharmacia Company, Sweden) was used according to the manufacturer’s directions. The lower limit of detection (LLD) of serum T-IgE, aspergillus specific IgE, and aspergillus specific IgG is 2 kU/L, 0.01 kUA/L, and 2 mg/L respectively. It was determined as elevation if serum T-IgE > 60 kU/L, *A. fumigatus* specific IgE >0.35 kUA/L, and *A. fumigatus* specific IgG > 40 mg/L.

### Skin prick test (SPT)

SPT was performed by injecting inhaled allergen extract of *A. fumigates* (Allergopharma Company, Germany) intradermally in the forearm. Histamine dihydrochloride and normal saline served as the positive and the negative control respectively.

### Peripheral eosinophil count

The total leukocyte count and the percentage of eosinophils were initially determined using an auto-analyzer. As for the patients with serum T-IgE ≥1000 kU/L, the percentage of eosinophils was ascertained by counting and classifying 100 WBCs on a peripheral blood smear.

### HRCT of the chest and determination of central bronchiectasis

HRCT of the chest was performed using a 64-row, multiple-detector CT scanner (Philips Company, Netherland). A radiologist blinded to the clinical and laboratory data reviewed the CT scans. The diagnosis of bronchiectasis on chest HRCT was made if bronchial wall thickening was present with the ratio of the diameter of bronchus to that of the accompanying pulmonary artery being more than 1.1 (signet ring sign), or the lack of tapering of bronchi (tramline sign). Central bronchiectasis was defined as the presence of bronchiectasis in the central two thirds of the lung field [13,14].

### Pulmonary function test

Spirometry (JAEGER, MasterScreen-body + diffusion + APS, Germany) was performed to determine the lung function measurements and bronchodilator reversibility. Post-bronchodilator FEV1/FVC% and FEV1 was measured 15 min after inhalation of 400 μg salbutamol.

### Statistical analysis

All statistical analyses were performed using a statistical software package (Statistics Package for the Social Sciences, SPSS 17.0, Inc., Chicago, IL, USA). Data are expressed as the mean ± SD (standard deviation). Comparisons of continuous data between two groups were performed by T-test (for normal distribution parameters) and Mann–Whitney U test (for abnormal distribution parameters). Comparisons of continuous data among three groups were performed by ANOVA test (for normal distribution) or Kruskal Wallis test (for abnormal distribution). Categorical variables between different groups were analyzed by χ^2^ test. Spearman Correlations were used for correlation analysis. *P* values less than 0.05 were considered as statistically significant. As for multiple comparisons among three groups (for χ^2^ test and Kruskal Wallis Test), *P* values less than 0.017 were considered as statistically significant.

## Results

### Baseline characteristics of the study population

The 273 patients with COPD consisted of 174 males (63%) and 99 females (37%) with a mean age of 77 (51 ~ 90) years. The baseline characteristics of the patients are shown in Table 1. The majority of the patients were smokers (82%) and some patients were exposed to biomass fuel (15%) or occupational dusts (14%). As for the symptoms, the average time of history of chronic cough/expectoration and that of exertional dyspnea were 19 years and 6.1 years respectively. The ratio of duration of dyspnea to that of chronic cough/expectoration was 0.32 on average. Most of the patients (78%) reported a history of wheezing. The majority of the patients (73.2%) belonged to GOLD stage III and IV by the spirometry criteria.

**Table 1:** **Baseline characteristics of the study population**

**Variables**	**Patients with COPD(273 cases)**
Male, n(%)	174(63)
Age (yr)	77(8,51 ~ 90)
Risk factors	
Smokers, n(%)	224(82)
Smoking index (pack-yr)	43(23,10 ~ 120)
Biomass fuel exposure, n(%)	42(15)
Biomass fuel exposure history (yr)	27(7,10 ~ 40)
Occupational dust exposure, n(%)	37(14)
Occupational dust exposure history (yr)	25(10,10 ~ 40)
History of chronic cough/sputum (yr)	19(12,2 ~ 50)
History of dyspnea (yr)	6.1(5.0,0 ~ 20)
Ratio of dyspnea history to chronic cough/sputum history	0.32(0.21,0 ~ 1)
Occurrence of wheezing, n(%)	214(78)
FEV1/FVC%	46(11,22 ~ 69)
FEV1%predicted	41(13,14 ~ 79)
GOLD stage	
Stage II, n(%)	73(26.8)
Stage III, n(%)	131(47.9)
Stage IV, n(%)	69(25.3)
Serum T-IgE (kU/L)	401(916,2 ~ 7420)

Continuous data expressed as mean (standard deviation, range).

Categorical data expressed as number (percentage).

*Definition of abbreviations*: FEV1: Forced expiratory volume in 1 second, FVC: Forced vital capacity.

### Prevalence of serum T-IgE elevation in the control population

Since serum T-IgE level may be influenced by other factors besides allergy, such as genetic factors, serum level of T-IgE was detected in a control population of 150 subjects [percentage of male: 64% (96), average age: 75 (9, 51 ~ 95), percentage of smokers: 78% (117)]. No statistical difference was shown in gender (p = 0.96), age (p = 0.08), and smoking history (p = 0.31) of the control population when compared with those of COPD patients. Elevated T-IgE level was found in 18.0% of the population (27/150), and the average level of T-IgE was 48(71,2 ~ 340) kU/L.

### Prevalence of serum T-IgE elevation, *A. Fumigatus* sensitization and ABPA in patients with COPD

**Figure 2:**
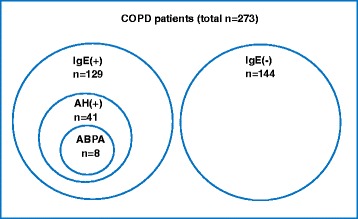
**Prevalence of increased serum IgE,** ***Aspergillus*** **hypersensitivity, and ABPA in patients with COPD.** The prevalence of increased serum IgE, *Aspergillus* hypersensitivity, and allergic bronchopulmonary aspergillosis in patients with COPD was shown with a disproportional Venn diagram. *Definition of abbreviations*: IgE(+): increased serum IgE, IgE(−): normal serum IgE, AH: *Aspergillus* hypersensitivity, ABPA: allergic brochopulmonary aspergillosis.

The prevalence of increased serum T-IgE, *A. Fumigatus* hypersensitivity and ABPA was shown in Figure 2. Elevated serum T-IgE was found in 47.3% (129/273) of the COPD patients, and the average level of T-IgE was 401(916,2 ~ 7420) kU/L (see Table 1), both of which were significantly higher when compared with those of the control population (p < 0.001, and p < 0.001, respectively). *A. Fumigatus* hypersensitivity was confirmed in 15.0% (41/273) patients, all of whom had an increased level of serum T-IgE, i.e. 31.8% of the COPD patients with elevated serum T-IgE (41/129) were hypersensitive to *A. Fumigatus* (see Table 2). Eight patients (2.9%, 8/273) met the diagnostic criteria for combined COPD and ABPA, 5 (1.8%, 5/273) with ABPA-CB and 3 (1.0%, 3/273) with ABPA-S. Among COPD patients with elevated serum T-IgE, the occurrence of ABPA, ABPA-CB and ABPA-S was 6.2% (8/129), 3.9% (5/129) and 2.3% (3/129) respectively. Among COPD patients with *A. Fumigatus* hypersensitivity, the prevalence of ABPA, ABPA-CB and ABPA-S was 19.5% (8/41), 12.2% (5/41) and 7.3% (3/41) respectively. The clinical data of the 8 patients with combined COPD and ABPA were shown in Table 3.

**Table 2:** **Comparison of participant characteristics between two COPD groups**

**Varibles**	**Group with elevated serum T-IgE (129 cases)**	**Group with normal serum T-IgE (144 cases)**	***P*** **value**
Male, n(%)	89(68.9)	85(59.0)	0.09
Age	76(9.2,51 ~ 90)	77(6.2,58 ~ 89)	0.12
Non smokers, n(%)	20(15.5)	29(20.1)	0.32
Smoking index of smokers (pack-yr)	43(22,10 ~ 100)	42(23,10 ~ 120)	0.52
History of chronic cough/sputum (yr)	19(11,2 ~ 45)	20(12,2 ~ 50)	0.42
History of dyspnea (yr)	8.0(5.2,0.5 ~ 20)	4.3(4.2,0 ~ 20)	<0.01
Ratio of dyspnea history to chronic cough/sputum history	0.47(0.19,0.07 ~ 1)	0.20(0.15,0 ~ 0.67)	<0.01
Occurrence of wheezing, n (%)	113(87.6)	101(70.8)	<0.01
FEV1/FVC%	43(11,23 ~ 67)	49(12,22 ~ 69)	<0.01
FEV1%predicted	36(11,14 ~ 68)	45(14,20 ~ 79)	<0.01
GOLD stage			<0.01
Stage II, n(%)	20(15.5)	53(36.8)	
Stage III, n(%)	62(48.1)	69(47.9)	
Stage IV, n(%)	47(36.4)	22(15.3)	
Serum T-IgE (kU/L)	818(1205,69 ~ 7420)	28(20,2 ~ 60)	
AH, n(%)	41(31.8)	0(0)	
ABPA, n(%)	8(6.2)	0(0)	

Continuous data expressed as mean (standard deviation, range).

Categorical data expressed as number (percentage).

*Definition of abbreviations*: AH: *Aspergillus fumigatus* hypersensitivity, ABPA: Allergic bronchopulmonary aspergillosis, FEV1: Forced expiratory volume in 1 second, FVC: Forced vital capacity.

**Table 3:** **Clinical characteristics of COPD patients complicated with ABPA**

	**Case 1**	**Case 2**	**Case 3**	**Case 4**	**Case 5**	**Case 6**	**Case 7**	**Case 8**
**Gender**	**Male**	**Female**	**Male**	**Male**	**Female**	**Female**	**Male**	**Male**
Age (yr)	78	73	81	57	73	80	54	67
Chronic cough/sputum history (yr)	30	30	25	8	30	20	3	27
Dyspnea history (yr)	10	20	15	5	15	15	3	20
Ratio of dyspnea history to chronic cough/sputum history	0.33	0.67	0.60	0.63	0.50	0.75	1.00	0.74
Occurrence of wheezing	Yes	Yes	Yes	Yes	Yes	Yes	Yes	Yes
Eosinophil of peripheral blood (%)	11.7	8.6	7.5	8.0	5.1	5.0	16.3	5.5
Serum T-IgE (kU/L)	7420	1239	5000	2049	1187	2272	1279	1160
Serum aspergillus specific IgE (kUA/L)	4.89	62.3	6.91	2.07	3.58	2.30	22	0.70
Serum aspergillus specific IgG (mg/L)	121	ND	102	ND	ND	92	ND	68
SPT (grade)	(++)	(+++)	(++)	(++)	(++++)	(++)	(++)	(++)
Chest HRCT								
Central bronchiectasis	Yes	No	Yes	No	Yes	Yes	Yes	No
Mucoid impaction	No	No	Yes	No	No	No	No	No
Pulmonary opacities	Yes	Yes	Yes	Yes	Yes	Yes	Yes	Yes
Lung function (postbronchodilator)								
FEV1/FVC(%)	31	30	30	23	35	67	42	42
FEV1%predicted	35	26	25	14	39	36	30	23
Type of ABPA	CB	S	CB	S	CB	CB	CB	S

*Definition of abbreviations*: ND: Not detected, SPT: Skin prick test, HRCT: high resolution computed tomography, T-IgE: Total IgE, FEV1: Forced expiratory volume in 1 second, FVC: Forced vital capacity, CB: Central bronchiectasis, S: Seropositive.

**Table 4:** **Comparison of participant characteristics between two COPD groups in 142 males with smoking**

**Variables**	**Group with elevated serum T-IgE (75 cases)**	**Group with normal serum T-IgE (67 cases)**	***P*** **value**
Age (yr)	75(10,54 ~ 90)	77(7,58 ~ 89)	0.19
Smoking index (pack-yr)	48(22,10 ~ 100)	48(25,10 ~ 120)	0.85
Chronic cough/sputum history (yr)	18(13,2 ~ 45)	19(12,2 ~ 40)	0.56
Dyspnea history (yr)	7.7(5.3,1 ~ 20)	3.7(3.7,0 ~ 20)	<0.01
Ratio of dyspnea history to chronic cough/sputum history	0.49(0.20,0.07 ~ 1)	0.19(0.14,0 ~ 0.5)	<0.01
Occurrence of wheezing, n(%)	65(87)	45(67)	<0.01
FEV1/FVC%	44(11,23 ~ 67)	48(11,22 ~ 69)	0.03
FEV1%predicted	37(12,14 ~ 68)	44(14,20 ~ 79)	<0.01
GOLD stage			<0.01
Stage II, n(%)	12(16.0)	22(32.8)	
Stage III, n(%)	37(49.3)	35(52.2)	
Stage IV, n(%)	26(34.7)	10(14.9)	

Continuous data expressed as mean (standard deviation, range).

Categorical data expressed as number (percentage).

*Definition of abbreviations*: FEV1: Forced expiratory volume in 1 second, FVC: Forced vital capacity.

**Table 5:** **Comparison of participant characteristics among three groups in 142 males with smoking**

**Variables**	***Af*** **(+)/IgE(+) (24 cases)**	***Af*** **(−)/IgE(+) (51 cases)**	***Af*** **(−)/IgE(−) (67 cases)**
Age (yr)	76(11,54 ~ 90)	74(10,55 ~ 88)	77(7,58 ~ 89)
Smoking index (pack-yr)	47(20,10 ~ 90)	48(23,10 ~ 100)	48(25,10 ~ 120)
Chronic cough/sputum history (yr)	19(13,2 ~ 40)	17(13,2 ~ 45)	19(12,2 ~ 40)
Dyspnea history (yr)^♦^	8.8(5.6,1 ~ 20)*	7.1(5.2,1 ~ 20)*	3.7(3.7,0 ~ 20)
Ratio of dyspnea history to chronic cough/sputum history^♦^	0.52(0.22,0.2 ~ 1)*	0.47(0.19,0.07 ~ 1)*	0.19(0.14,0 ~ 0.5)
Occurrence of wheezing, n(%)	21(88)	44(86)	45(67)
FEV1/FVC%	44(13,23 ~ 67)	43(10,28 ~ 64)	48(11,22 ~ 69)
FEV1%predicted^♦^	37(14,14 ~ 68)^#^	37(11,19 ~ 64)*	44(14,20 ~ 79)
GOLD stage^♣^			
Stage II, n(%)	3(12.5)	9(17.6)	22(32.8)
Stage III, n(%)	12(50.0)	25(49.0)	35(52.2)
Stage IV, n(%)	9(37.5)	17(33.3)	10(14.9)

*Compared with *Af*(−)/IgE(−), *P* < 0.01.

^#^Compared with *Af*(−)/IgE(−),*P* <0.05.

♦Overall comparison among three groups, *P* <0.01.

♣Overall comparison among three groups, *P* <0.05.

*Definition of abbreviations*:

*Af*(+)/IgE(+): *Aspergillus fumigatus* hypersensitivity with elevated serum total IgE.

*Af*(−)/IgE(+): no *Aspergillus fumigatus* hypersensitivity but with elevated serum total IgE.

Af(−)/IgE(−): no *Aspergillus fumigatus* hypersensitivity with normal serum total IgE.

FEV1: Forced expiratory volume in 1 second, FVC: Forced vital capacity.

### Comparison between COPD patients with elevated serum T-IgE and those with normal serum T-IgE

There was no significant difference in gender, age, the percentage of smokers, smoking index of the smokers and history of chronic cough/expectoration between the group with elevated T-IgE (n = 129) and that with normal T-IgE (n = 144). Compared with the normal IgE group, patients with elevated T-IgE had a longer history of exertional dyspnea, an earlier onset of dyspnea after chronic cough/expectoration, and were more likely to wheeze. COPD patients with elevated serum T-IgE also showed worse pulmonary functions, as demonstrated by lower values of FEV1/FVC% and FEV1%predicted, and by more severe GOLD staging (see Table 2).

Among the male patients with smoking as the risk factor, no significant difference was found in age, smoking index and history of chronic cough/expectoration between the group with elevated T-IgE (n = 75) and that with normal T-IgE (n = 67), but patients with increased serum T-lgE were more likely to wheeze, had a longer history of dyspnea and earlier onset of dyspnea on the background of chronic cough/expectoration history. They also had a worse pulmonary function (see Table 4).

*A. Fumigatus* is one of the major specific allergens believed to have an impact on chronic airway inflammatory diseases such as asthma and COPD. Therefore, we further divided the 75 male patients with elevated serum T-IgE into two subgroups, one with *A. Fumigatus* hypersensitivity (AH) [*Af*(+)/IgE(+), n = 24] and another without AH [*Af*(−)/IgE(+), n = 51], and compared the clinical data of the *Af*(+)/IgE(+) subgroup, the *Af*(−)/IgE(+) subgroup, and the group with normal serum T-IgE [*Af*(−)/IgE(−), n = 67]. There was no significant difference in age, smoking index and history of chronic cough/expectoration. When compared with the *Af*(−)/IgE(−) group, both the *Af*(+)/IgE(+) subgroup and the *Af*(−)/IgE(+) subgroup showed longer history of dyspnea, an earlier onset of dyspnea and a worse status of lung function (FEV1%predicted). The severity by GOLD stages and the prevalence of wheezing were significantly different among the three groups, but no statistical difference was found between any two groups. There was also no difference in the clinical data between the Af(+)/IgE(+) subgroup and the *Af*(−)/IgE(+) subgroup (see Table 5).

### Correlation of serum T-IgE with dyspnea history and pulmonary function measurements

**Figure 3:**
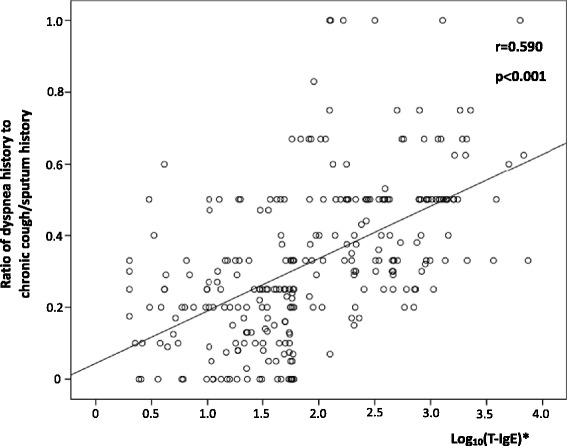
**Relationship between T-IgE and ratio of dyspnea history to chronic cough/sputum history in 273 patients.** *Correlation between log_10_ (T-IgE) and ratio of dyspnea history to chronic cough/sputum history.

**Figure 4:**
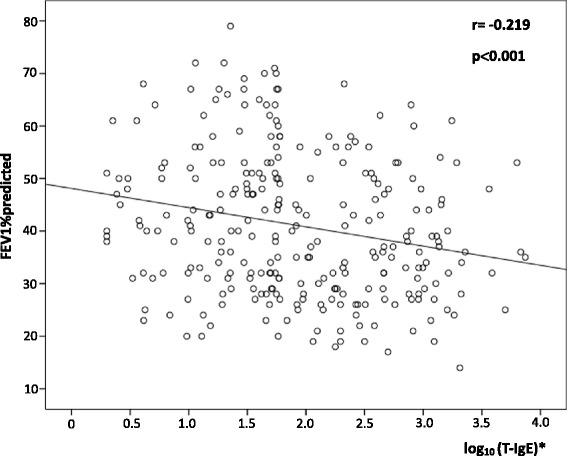
**Relationship between T-IgE and FEV1%predicted in 273 patients.** *Correlation between log_10_ (T-IgE) and FEV1%predicted.

As there were significant differences in history of exertional dyspnea and pulmonary function measurements (FEV1/FVC% and FEV1%predicted) between patients with elevated serum T-IgE and those with normal T-IgE, we performed correlation analysis of serum T-IgE with these parameters. Serum T-IgE was shown to be correlated positively with the time length of dyspnea history (r = 0.401, *P*< 0.001), and the ratio of duration of dyspnea to that of chronic cough/expectoration (r = 0.590, *P*< 0.001) (Figure 3), but negatively with FEV1/FVC% (r = −0.194, *P* = 0.001), and FEV1%predicted (r = −0.219, *P*< 0.001) (Figure 4).

## Discussion

Previous studies have demonstrated that allergy was associated with decreased lung function in general population, smokers and mild COPD patients [15-18]. However, data on the prevalence of allergy in a larger sample size of patients with COPD and its association with clinical characteristics are lacking. Our study showed for the first time to our knowledge that increased serum total IgE, a sensitive marker for allergy, was prevalent in patients with COPD and associated with earlier onset and longer duration of dyspnea in the course of the disease and more severe lung function impairment. Furthermore, *Aspergillus* sensitization was common in COPD patients with increased levels of IgE, and some patients were confirmed to have combined COPD and ABPA.

The increased serum level of T-IgE may result from allergic inflammation at other parts of the host, and may be associated with genetic factors and even smoking history [19,20]. In our study, the increased level of serum IgE was considered to mostly reflect the allergic inflammation of the airways. Since firstly, the prevalence and the level of IgE elevation were much lower in the control population when compared with those of the COPD patients. Secondly, no allergic inflammation of other systems such as the skin was noted, and parasite infection was excluded in our study subjects according to history taking, physical examination and accessory examinations. Thirdly, all the patients with positive *Aspergillus*-specific IgE showed elevated serum T-IgE, and no positive result of *Aspergillus*-specific IgE was found in patients with normal serum T-IgE, suggesting a high consistency between serum T-IgE and airborne allergen specific IgE in our cohort. The results demonstrated that the prevalence of elevated serum T-IgE was 47.3% (129/273) in patients with COPD, implying that even among COPD patients without obvious atopy, hypersensitive inflammation of the lower airways may exist, probably representing the real proportion of the allergic phenotype in patients with COPD, a much higher prevalence as compared to those reported by Jamieson et al. [2] and Bafadhel et al. [5]. The reason for the difference may be easily understood, since firstly, the criteria for “allergy” were different. Just as mentioned above, measurement of serum T-IgE may be more sensitive than results of specific IgE and SPT for the common allergens. Allergic sensitization in adults is generally believed to be stable over time [2]. We also observed that several results of serum IgE of the same patient measured at different time were usually consistent [data not shown]. However, we could not exclude the possibility that some patients might have a mild increase of serum T-IgE only for once, because we did not follow the IgE changes in all the patients.

*Aspergillus* sensitization and occurrence of ABPA in COPD patients are receiving considerable interests. Agarwal et al. reported that the prevalence of *Aspergillus* sensitization and ABPA in a cohort of COPD patients without atopy was 8.5% and 1% respectively [4], while in the study by Bafadhel et al. the prevalence of *Aspergillus* sensitization in COPD was 13% [5]. Our study showed that the prevalence of *Aspergillus* sensitization in patients with COPD was 15%, similar to that reported by Bafadhel et al., but higher than that by Agarwal et al. Besides the different study subjects, different methods to determine *Aspergillus* sensitization may be a main reason for the discrepancy, because detection of specific IgE (used in our study) has been shown to be more sensitive than SPT (used by Agarwal) to identify *Aspergillus* sensitization [12,21,22]. We showed that the prevalence of ABPA in COPD patients, in those with elevated serum IgE, and in those with *Aspergillus* sensitization was 2.9%, 6.2%, and 19.5% respectively, indicating that the occurrence of ABPA in COPD was lower than that in asthma [3,19]. The reason for different prevalence of ABPA reported by us and Agarwal et al. may be explained by different disease severity and demographic features of the patients. The GOLD stages of the patients in our study were more advanced, and they were older (76 years vs. 57 years), had a longer course of disease(19 years vs. 6.1 years) , and there were more females (37% vs. 10%).

We found no significant difference in the history length of chronic cough/expectoration between patients with elevated serum IgE and those with normal serum IgE, but the former group was more likely to wheeze, which was similar to the result of Jamieson et al. [2]. Because exertional dyspnea is the hallmark symptom of COPD and closely associated with quality of life of the patients, we examined the potential implication of IgE in the development and progression of this symptom. We showed that COPD patients with elevated serum IgE suffered for a longer history of dyspnea, had an earlier onset of dyspnea on the background of chronic cough/sputum, and had a worse lung function. To exclude the effects of gender and different risk factor exposures, we further compared the symptoms and lung function only in male patients with smoking as the risk factor, and reached the same results. This suggested that serum IgE level might, to some degree, have an effect on the progression of COPD, or could be a useful marker to reflect the severity of disease. In Jamieson’s study, no significant difference in lung function (FEV1%predicted) was found between the “allergic phenotype” and the “nonallergic phenotype” of COPD patients [2]. We demonstrated that compared with COPD patients with normal serum IgE, patients with elevated serum IgE had a worse lung function (FEV1/FVC and FEV1%predicted). The reason for the discrepancy could be as follows: firstly, the diagnostic criteria of COPD were stricter in our study, while some patients with asthma or combined asthma and COPD could be involved in Jamieson’s study [2]. Secondly, the criteria for “allergic” were different. The patients allergic to allergens other than what was detected in Jamieson’s study might be identified as “nonallergic phenotype”. Thirdly, the sample size of the two studies was different (273 cases vs. 77 cases). Finally, our patients were older (75 years vs. 69 years). It is believed that the impacts of allergy on lung function may be increasing with age [23].

IgE is not only a sensitive marker for the presence of allergic inflammation, but also correlated positively with airway inflammation and remodeling in asthma [6-8]. Zedan et al. reported that the shortness of breath (SOB) group of asthma patients showed significant increase in total serum IgE when compared with both the cough and the wheezy groups [24]. Since asthma and COPD shared some pathological characteristics [25], we speculated that elevated serum T-IgE might promote the airway inflammation and remodeling, and then resulted in more serious symptoms and worse status of lung function. As expected, our results showed that the serum IgE level was correlated with the length of dyspnea history and lung function parameters (FEV1/FVC and FEV1%predicted). It was suggested by Vroling et al. [26] and Tsai et al. [27] that the effect of allergy on airway inflammation in COPD was due to locally enhanced inflammation.

Previous studies have shown that lung function is more severely impaired in COPD patients with *Aspergillus* sensitization [5]. Our study confirmed that significant difference existed in dyspnea history and lung function (FEV1%predicted) between patients with *Aspergillus* sensitization and those with normal serum IgE. However, no significant difference in dyspnea history and lung function was found between patients with *Aspergillus* sensitization and those with elevated serum IgE but without *Aspergillus* sensitization. This suggests that allergens other than *Aspergillus* may have similar effects on COPD, but further study is needed for a final conclusion.

COPD combined with ABPA is believed to be rare, although more cases were reported recently [28-30], possibly due to an increased awareness of this condition. In our patients, eight were confirmed to have combined COPD and ABPA, including five patients with ABPA-CB. It is noteworthy that all the eight patients with ABPA showed much worse lung function measurements, with FEV1%predicted ranging from 14 to 39. It is postulated that ABPA developing in patients with COPD may present with a slightly different clinical profile as compared to patients with asthma [29].

There are several limitations to our study. As a cross-sectional study, serum T-IgE and *Aspergillus* specific IgE were not followed in all the patients, although allergic sensitization in adults is generally believed to be stable. Because of the retrospective nature of the study, more measures of patient reported outcome (PRO) such as COPD Assessment Test (CAT), and history of exacerbations which might have an association with allergy in a proportion of patients, had not been collected and analyzed. We did not analyze the difference in long-term drug therapy, particularly inhaled corticosteroids in these patients. Medication use and compliance of the patients may affect symptoms and lung function, although it is possible to have a similar effect on level of serum IgE [24]. Finally, the time interval between an exacerbation and the enrollment of the patients to our study needs to be mentioned. The recovery time of an exacerbation varies considerably among patients, although a number of studies on stable COPD recruited patients without an exacerbation in the previous 4 weeks [31-33]. One earlier study showed that the median time to recovery was 6 d (1–14) for peak expiratory flow rate (PEFR) and 7d (4–14) for symptoms, but recovery of PEFR to baseline values was complete in only 75.2% of exacerbations at 35d [34]. In our study, 18% (51/273) of the COPD patients were enrolled within 2 ~ 4 weeks after an exacerbation, when the lung functions were still recovering at least in some of the patients. We performed an analysis after exclusion of this small proportion of patients, and reached the same results as those of the whole population (data not shown). Therefore, we believe that the time interval we chose to enroll our patients had little effect on the association of IgE with symptoms and lung function measures.

## Conclusions

In conclusion, to our knowledge, this is the first study to investigate serum T-IgE level in patients with COPD and its association with the symptoms and lung function. It is also a study of the largest sample size for the investigation of the prevalence of *Aspergillus* sensitization in COPD patients. There was a high prevalence of elevated serum T-IgE in patients with COPD but without obvious atopy. Serum T-IgE level was found to be associated with symptoms such as dyspnea and impairment of lung function. Recurrent exacerbation of COPD is believed to accelerate disease progression and impairment of pulmonary function, and related to increased disability and mortality [35,36]. Acute exacerbation of COPD can be precipitated by several factors, but the most common causes appear to be viral or bacterial respiratory infections [37]. However, the cause of about one-third of severe exacerbations of COPD cannot be identified [37]. Because the allergic phenotype of COPD was shown to have an increased risk of exacerbations [2], whether airway allergy plays a role in the susceptibility to, or is an unidentified trigger for exacerbation is worth further speculating. Finally, longitudinal studies may also be needed to examine the potential role of allergy in disease expression or progression of COPD.

## Abbreviations

COPD: Chronic obstructive pulmonary disease
IgE: Immunoglobulin E
T-IgE: Total IgE
*A. Fumigatus*: *Aspergillus Fumigatus*
AH: *Aspergillus* hypersensitivity
ABPA: Allergic bronchopulmonary aspergillosis
ABPA-CB: ABPA with central bronchiectasis
ABPA-S: Seropositive ABPA
HRCT: High resolution computed tomography
mMRC: Modified Medical Research Council Questionnaire
FEV1: Forced expiratory volume in 1 second
FVC: Forced vital capacity
GOLD: The Global Initiative For Chronic Obstructive Lung Disease
LLD: Lower limit of detection
PRO: Patient reported outcome
CAT: COPD Assessment Test
